# Enhancing therapeutic reasoning: key insights and recommendations for education in prescribing

**DOI:** 10.1186/s12909-024-06310-4

**Published:** 2024-11-26

**Authors:** Mariëlle G. Hartjes, Milan C. Richir, Yoann Cazaubon, Erik M. Donker, Ellen van Leeuwen, Robert Likic, Yves-Marie Pers, Joost D. Piët, Fabrizio De Ponti, Walter Raasch, Floor van Rosse, Jitka Rychlícková, Emilio J. Sanz, Markus Schwaninger, Susanna M. Wallerstedt, Theo P. G. M. de Vries, Michiel A. van Agtmael, Jelle Tichelaar, On behalf of the EACPT Education Working group

**Affiliations:** 1 Department of Internal Medicine, Unit Pharmacotherapy, Amsterdam UMC, Vrije Universiteit, De Boelelaan 1117, 1081 HV Amsterdam, The Netherlands; 2 Research and Expertise Centre in Pharmacotherapy Education (RECIPE), De Boelelaan 1117, 1081 HV Amsterdam, The Netherlands; 3 Interprofessional Collaboration and Medication Safety, Faculty of Health, Sports and Social Work, InHolland University of Applied Sciences, Pina Bauschplein 4, 1095PN Amsterdam, The Netherlands; 4 Department of Surgery, University Medical Center Utrecht, Heidelberglaan 100, 3584 CX Utrecht, The Netherlands; 5 Department of Pharmacology, Montpellier University Hospital, Avenue du Doyen Gaston Giraud, 34090 Montpellier, France; 6 Pathogenesis and Control of Chronic and Emerging Infections (PCCEI), INSERM, University Montpellier, 34090 Montpellier, France; 7 Department of Fundamental and Applied Medical Sciences, Unit of Clinical Pharmacology, Ghent University, C. Heymanslaan 10, 9000 Ghent, Belgium; 8 Unit of Clinical Pharmacology, Department of Internal Medicine, University Hospital Centre Zagreb and University of Zagreb School of Medicine, 12 Kišpatićeva St, 10 000, Zagreb, Croatia; 9 IRMB, University Montpellier, INSERM, CHU Montpellier, Montpellier, France; 10 Clinical Immunology and Osteoarticular Diseases Therapeutic Unit, Lapeyronie University Hospital, Montpellier, France; 11 Department of Medical and Surgical Sciences, Pharmacology Unit, Alma Mater Studiorum, University of Bologna, Via Zamboni 33, 40126 Bologna, Italy; 12 Institute of Experimental and Clinical Pharmacology and Toxicology, University of Lübeck, Lübeck, Germany; 13 Department of Hospital Pharmacy, University Medical Center Rotterdam, MC Rotterdam, The Netherlands; 14 Department of Pharmacology, Faculty of Medicine, Masaryk University, Brno, Czech Republic; 15 School of Health Science, Universidad de La Laguna, and Hospital Universitario de Canarias (SCS), Santa Cruz de Tenerife, Calle Padre Herrera, S/N, 38200 La Laguna Tenerife, Spain; 16 Department of Pharmacology, Sahlgrenska Academy, University of Gothenburg, Gothenburg, Sweden

**Keywords:** Therapeutic reasoning, Medical decision making, Clinical pharmacology and therapeutics, Medical education, Management reasoning, Clinical competency

## Abstract

**Background:**

Despite efforts to improve undergraduate clinical pharmacology & therapeutics (CPT) education, prescribing errors are still made regularly. To improve CPT education and daily prescribing, it is crucial to understand how therapeutic reasoning works. Therefore, the aim of this study was to gain insight into the therapeutic reasoning process.

**Methods:**

A narrative literature review has been performed for literature on cognitive psychology and diagnostic and therapeutic reasoning.

**Results:**

Based on these insights, The European Model of Therapeutic Reasoning has been developed, building upon earlier models and insights from cognitive psychology. In this model, it can be assumed that when a diagnosis is made, a primary, automatic response as to what to prescribe arises based on pattern recognition via therapy scripts (type 1 thinking). At some point, this response may be evaluated by the reflective mind (using metacognition). If it is found to be incorrect or incomplete, an alternative response must be formulated through a slower, more analytical and deliberative process, known as type 2 thinking. Metacognition monitors the reasoning process and helps a person to form new therapy scripts after they have chosen an effective therapy. Experienced physicians have more and richer therapy scripts, mostly based on experience and enabling conditions, instead of textbook knowledge, and therefore their type 1 response is more often correct.

**Conclusion:**

Because of the important role of metacognition in therapeutic reasoning, more attention should be paid to metacognition in CPT education. Both trainees and teachers should be aware of the possibility to monitor and influence these cognitive processes. Further research is required to investigate the applicability of these insights and the adaptability of educational approaches to therapeutic reasoning.

## Background

A professor of Clinical Pharmacology recounted how a former student, now a practicing physician, told him she had forgotten most of what she learned, except for the “red warning light” that activates whenever she considers prescribing a medication. This triggers critical questions and initiates the process of selecting the best treatment for her patients. The professor was pleased with this response and frequently incorporated the red warning light into his teaching. In this review we describe what is now known about the process behind the ‘red warning light’, and what we can learn from it for education, clinical practice and further research. To clearly describe and understand this complicated cognitive process, we have included a model of the thinking process, and a realistic patient case for illustration.

Over the years, considerable effort has been invested in improving clinical pharmacology and therapeutics (CPT) education and assessment [1, 2]. These improvements have occurred at both local, national, European, and worldwide levels. Examples include joint assessments conducted both nationally and at a European level, harmonization of education systems and requirements, sharing of materials, and teach-the-teacher programs resulting from European and worldwide collaborations [3–10]. Despite these efforts, further steps still need to be taken to improve CPT education and, thereby, prescribing practices. This is because evidence shows that residents’ prescribing knowledge and skills can still be improved. Additionally, these skills do not seem to improve during their first year of clinical practice, and residents still make many errors [11, 12]. These errors may lead to patients’ harm, decreased quality of life, and increased healthcare costs [13, 14].

If prescribing were based solely on guidelines and knowledge, then improving the use and quality of guidelines should reduce the number of prescribing errors. Yet, this seems not to be the case – prescribing involves much more than following guidelines: it is a complex skill that needs to be developed by training and doing in different clinical scenarios [15]. Indeed, a prescriber must be aware of specific patient characteristics such as comorbidities and co-medication, the severity of the disease, drug characteristics and clinical context, and how these influence the choice of medication in order to establish the most appropriate treatment, and also taking patient’s preference into account. This requires a high level of the so-called therapeutic reasoning, which is a subset of management reasoning.

Developing therapeutic reasoning requires training and good examples well rooted in a clinical context. However, it is often observed in practice that when residents or students ask their supervisors to explain why a specific treatment has been chosen, they are often told that the supervisor follows the current guideline or has used this treatment for this condition for years with success, without being given detailed information about why it is the drug of choice in this specific patient. Because residents and students often rely on the examples set by their teachers or supervisors in clinical practice, they may prescribe the same medication for future patients without understanding why, merely repeating what they have seen [16]. For example, the supervisor may choose a drug on the basis of specific patient characteristics, but if the student or resident is not aware of this, he or she may prescribe the drug to future patients with the same disease but with other characteristics, potentially leading to prescribing errors. Moreover, the prescribing skills of experienced prescribers may become daily routine instead of an up-to-date skill, especially if they have prescribed the same drug for years, even when another (often newer) drug may be more appropriate. So, understanding the therapeutic reasoning process may help to improve the prescribing skills of both experienced and inexperienced prescribers.

While many studies and medical education have mainly focused on diagnostic reasoning as part of clinical reasoning, more needs to be learned about why physicians choose a specific therapy (i.e. therapeutic reasoning) [17]. Most existing models of therapeutic reasoning are mainly based on diagnostic reasoning, such as the models of Bissessur et al. [18], Denig et al. [19], and the World Health Organization (WHO) 6-step [20, 21]. However, although diagnostic and therapeutic reasoning are intricately linked, both have its own challenges, which will be discussed in this review. This raises the question of whether models developed for diagnostic reasoning can also be used for therapeutic reasoning. Therefore, therapeutic reasoning deserves a focus of its own. Consequently, a novel therapeutic approach grounded in therapeutic reasoning principles might add insights to the teaching of prescribing, thereby facilitating improved daily prescribing practices. Such a method can be based on contemporary research within cognitive psychology, a field in which reasoning is extensively studied and often provides insights which emphasize the complex nature of reasoning processes.

These new insights could serve as the foundation for renewing CPT education for both undergraduate and graduate prescribers. Therefore, the aim of this narrative review was to gather insights into the therapeutic reasoning process, identify knowledge gaps and provide a foundation for future research to improve CPT education and prescribing practices in clinical settings [22].

## Methods

Because the medical literature is not conclusive and the reasoning process has been widely studied in other disciplines, we decided to take a broad approach, by using a narrative review. The strength of a narrative review is that it seeks to identify what has been accomplished previously, allowing for consolidation, building on previous work and identifying knowledge gaps [23]. This method allows us to incorporate knowledge from other fields into the theories of therapeutic reasoning and to perform an additional search for extra information about relevant topics, such was whether theories from cognitive psychology are also incorporated within therapeutic reasoning. The PubMed, MEDLINE, EMBASE, PsycINFO, and CINAHL databases were searched for articles about therapeutic reasoning in English or Dutch, to gain a broad understanding of therapeutic reasoning among various healthcare professionals. The last search was performed on 15 November 2023. In addition, a more general search of studies about reasoning and decision making was performed, because of the profound insights into reasoning in general. The references of relevant articles were screened, using the snowball method. Search terms included (synonyms of) therapeutic reasoning, management reasoning, management decision making, therapy or management scripts, drug choice, and prescribing patterns. Studies involving both experienced and inexperienced prescribers and students of all professions with prescribing authority were included. Because of the extensiveness and complexity of the topic, the choice was made to also create a report, with a more extensive theoretical framework on this topic [22] on behalf of the EACPT education group and the Clinical Pharmacology and Therapeutics Teach the Teacher (CP4T) consortium. That report, in addition, extensively discusses the relevant knowledge of cognitive psychology and diagnostic reasoning. Here, we present a shorter review article, mainly focusing on the application of the insights from cognitive psychology and diagnostic reasoning to therapeutic reasoning. The complete report can be found on The European Open Platform for Prescribing Education (EurOP^2^E) [24].

## Therapeutic reasoning

### Introduction to therapeutic reasoning

Therapeutic reasoning concerns the process of establishing a management plan for an individual patient. A model and a patient case may be helpful in reading the findings of this review in the following paragraphs.

**Fig. 1:**
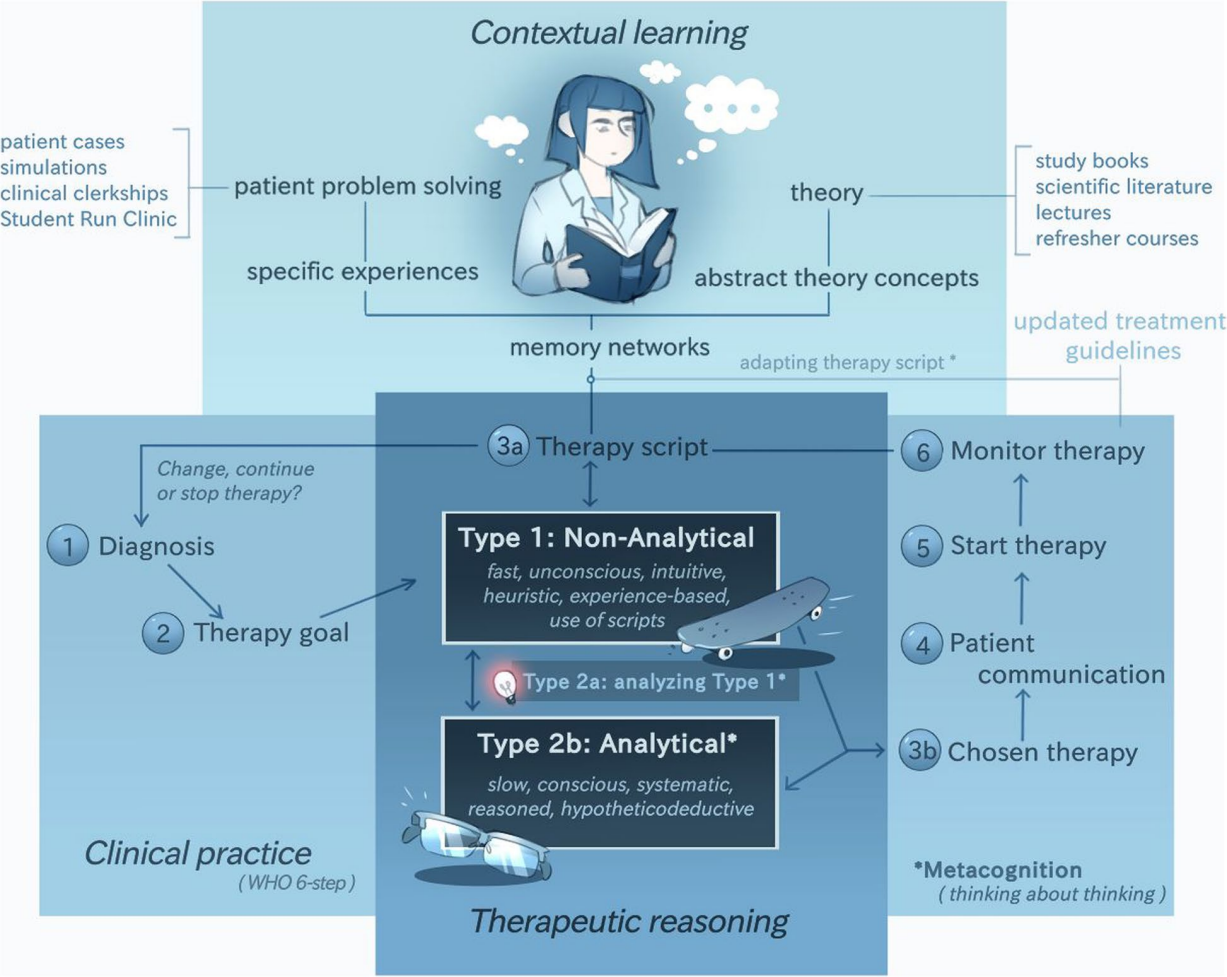
The European model of therapeutic reasoning within the context of clinical practice and contextual learning [21]

The European model of therapeutic reasoning (Fig. 1), which builds upon the earlier model by Tichelaar et al. [21], illustrates the process of therapeutic reasoning within the context of clinical practice and contextual learning. We have developed this model, based on the theory described in this narrative review. We will present our model first before describing the underlying theory.

The aim of therapeutic reasoning is establishing a management plan for an individual patient. When a patient problem is presented, type 1 thinking is always used. This type 1 thought can be concrete, which might directly lead to a therapy, or may be more abstract, for example “starting antibiotics”, requiring a type 2 analytical thinking process. The core aspect of this process is the therapy script. Such a script pops up from memory as soon as the therapy is thought about. For experienced physicians, it consists of (1) a small number of therapy options linked to a diagnosis, (2) some relevant patient characteristics and circumstances from previous experiences with similar patients, and (3) the results of previously initiated therapies. The therapy options result from evaluating different medications within and across medication groups, considering their respective advantages and disadvantages. A decision is then made in a fast, intuitive and heuristic manner (type 1 reasoning). Good prescribers then 'automatically' perform a check on that decision (with the help of metacognition; type 2a reasoning). If there is uncertainty about the correctness or completeness of the first decision (the red warning light), then a slower, conscious, more thorough and analytical reasoning follows (type 2b reasoning). On the basis the therapy chosen, therapy scripts can be developed which can be used in new situations. In an uncertain diagnosis the therapy choice can be used as diagnostic tool,e.g. seeing whether prednisone for presumed polymyalgia rheumatica or furosemide for presumed heart failure reduces symptoms, thus confirming the hypothesis.

This reasoning process belongs to the therapeutic part of a consultation in clinical practice. This is represented by the normative and circular WHO 6-step approach, where in step 1 the established (provisional) diagnosis is the starting point, and step 3 is the choice of therapy (3a: therapy script, 3b: the chosen therapy). Step 4, patient communication, which includes shared-decision making could also lead to an adapted therapy if necessary. In step 6, the established therapy for the patient involved is evaluated after some time. On the one hand, the result determines whether the therapy should be continued, adjusted or stopped. On the other hand, the result is (often unconsciously) added to the other experiences with this therapy, and the therapy script is adjusted if necessary (metacognition). Something similar happens if new treatment guidelines give reason to do so.

Developing therapy scripts starts already during undergraduate training, especially with contextual learning. The combination of studying theory and solving patient problems (simulated or in practice) leads to memory networks in the brain that result in illness and therapeutic scripts. Initially, these scripts are still small and insufficient to make a quick decision and therefore, inexperienced prescribers needs to use type 2 analytical reasoning more often.

The process of therapeutic reasoning can also be illustrated by a typical patient case (Table 1). Throughout this case, various terms regarding therapeutic reasoning are used, and will be explained in detail in the following sections. While the case is a helpful illustration, it is important to note that identifying specific types of reasoning, heuristics, or biases from individual cognitive episodes remains challenging. These concepts often serve as post hoc explanations and may not fully capture the complexity or predictive value of therapeutic decision-making in real-life scenarios.

**Table 1:** A typical patient case

Patient A. comes to the hospital with complaints of pain during urination and in the left flank. He is seen by the resident physician. The resident suspects that the patient has a urinary tract infection and wants to start antibiotics (***type 1 reasoning****)*. Since he has not been working in the emergency department for long, he decides to consult the guidelines to help him to find the best treatment *(****type 2 reasoning****)*. Initially, the resident considers whether the patient is also septic, whether there is tissue invasion, or if it is an uncomplicated cystitis. The patient's vital signs are stable, which makes urosepsis less likely. Given the flank pain, pyelonephritis seems possible, even though the patient does not have fever. To err on the side of caution, he decides to treat for pyelonephritis (***commission bias***) – a few weeks ago he had missed pyelonephritis and he does not want this to happen again (***with help of metacognition****)*. He then has the option to choose between oral and intravenous antibiotics. He has to decide treatment in a hurry because a new patient has arrived. He decides to start the same, intravenous treatment as for the previous patient with pyelonephritis *(****cognitive ease and last case bias****)*. He checks the formulary for any contraindications or interactions with the current medication
He then discusses the patient with the supervisor. The supervisor prefers to start with a different intravenous antibiotic *(****based on his therapy scripts****)*. When the resident asks why, the supervisor mentions his experience that patients tend to improve faster with this antibiotic, without being able to explain why *(****type 1 response, based on pattern recognition****)*. They decide together to administer this medication to the patient, and in the following days, the patient improves. The resident considers prescribing this medication in the future since it appears to work well *(****a new therapy script is formed with the help of metacognition****)*
He could switch to an oral antibiotic because the patient recovers well. Culture results show that the bacteria is sensitive to both cotrimoxazole and ciprofloxacin. The resident consults the guideline again and decides to start ciprofloxacin. He discusses it with his supervisor who prefers cotrimoxazole because following good antibiotic stewardship rules it is preferable to limit ciprofloxacin consumption (***factors influencing the reasoning process***). Eventually, the supervisor say the resident can choose from these options himself since both have their merits. The resident weighs some factors, such as interactions, costs, guidelines, side effects, and patient preference in his decision-making. Therefore, he also discuss the possibilities and wishes with the patient ***(shared decision making)***. The resident finally decides to prescribe ciprofloxacin because it has fewer side effects and interactions in this patient

To understand how the resident in this example arrives at a management plan for this patient, it is essential to delve into the theory of therapeutic reasoning combining this with the theories in cognitive psychology and diagnostic reasoning.

### Differences between therapeutic and diagnostic reasoning

It is often assumed that the therapeutic reasoning process is the same as that for diagnostic reasoning, both are closely related, can influence each other and are necessary for providing good care. Although there are similarities between these types of reasoning, like the probabilistic thinking and the availability of several options, there are some important differences. First of all, the patient is often actively involved in therapeutic reasoning, unlike in diagnostic reasoning. The diagnostic process is independent of patient preferences, practical constraints such as availability, and often starts with one working diagnosis at a specific time. In contrast, therapeutic reasoning also includes shared decision making and monitoring and patient and system preferences can play an important role. Management reasoning require ongoing monitoring and frequent adjustments. It involves a dynamic interplay among people, systems, setting and competing priorities, making this complex and “situated” and has unavoidable uncertainties [25].

### Theories of reasoning

#### Type 1 and type 2 thinking

Various cognitive psychologists have developed different theories about reasoning, and while these theories are broadly similar, there are some distinctions between them [26–31]. One of the most widely accepted theories of reasoning in decision-making is Kahneman's dual process theory [32]. Although Kahneman's model is the most well-known, similar models have been proposed earlier. In this article, we have chosen to focus on Kahneman’s theory because it is the most recognizable. Additionally, our new model also incorporates influences from other cognitive psychologists, drawing on a range of insights to provide a more comprehensive perspective. Both reasoning in general and clinical reasoning, which consists of both diagnostic and therapeutic reasoning, can be conceptualized in terms of type 1 versus type 2 thinking, wherein individuals can switch between the different types of thinking as needed [32]. This is also demonstrated in the case report described above, where a distinction is made between the primary, automatic response (type 1) and the more analytical response (type 2). The highlights of these theories will be described, applied to the context of therapeutic reasoning. Therapeutic reasoning often starts when the patient is diagnosed, but a physician can switch between reasoning about the diagnosis and the management and reflect on this during the reasoning process [33].

Type 1 thinking is automatic, associative, rapid, non-analytical thinking largely based on pattern recognition [34]. It occurs as soon as one is confronted with a problem. This mode of thinking requires minimal working memory, making it easier to apply [32]. Based on factors such as experience, it may lead to a sound decision, or it may be necessary to further critically evaluate it. This was also shown in the case report, where the resident knew that he should start antibiotics, but he was not certain about which one. In this case, a more analytical approach is necessary, which can be achieved through type 2 thinking.

Type 2 thinking is an analytical form of thinking in which multiple options are carefully considered and is slower than type 1 thinking [28]. In the case report, the resident decided to use guidelines and a formulary to make a considered choice of treatment. According to Stanovich, a psychologist, analytical thinking can be subdivided into two parts: reflective (type 2a) and algorithmic (type 2b) thinking [35]. Reflective thinking is necessary to recognize when a type 1 thought is insufficient and there is a need to switch to algorithmic thinking [35]. This reflective thinking is not only described by Stanovich, but also by Evans and Houdé, who refer to it as 'type 3 thinking [30, 31]. This phenomenon was also described by the “red warning light”, where the professor in the beginning referred to. For example in the case above, where the resident realizes that he needs to read the guideline for the most appropriate treatment, because he is not sure about the treatment. A student or physician needs to be able to recognize when a choice does not quite fit the current situation (conflict detection) and needs sufficient working memory for controlled thinking processes to accomplish the needed reasoning, which can be challenging during a busy shift with little sleep and many stimuli [36].

Once a conflict or complex situation has been recognized through reflective (type 2a thinking) (for example, due to insufficient knowledge or based on new patient characteristics), algorithmic thinking is activated (type 2b) [26–28, 30, 35]. This is a form of hypothetico-deductive reasoning, where various options are systematically weighed until the best one emerges with the help of probabilistic thinking [37–39]. However, achieving an optimal solution requires individuals to have the appropriate motivation, a sound understanding of reasoning principles, and sufficient cognitive capacity. Without these, errors are likely to occur [40]. As seen in the described case, numerous considerations have to be borne in mind, for example, patient-related factors (such as the severity of the illness and the potential of emerging new diseases/conditions), practice-related factors (such as personal experience), medication-related factors (such as side effects and interactions), information-related factors (such as guidelines), and education-related factors (such as the supervisor's example) [16, 19, 41–43]. All these factors need to be evaluated by the prescriber, but little is known about how this weighing process is done. The importance of these factors may differ per situation. Because of the complexity of prescribing medicines, a multidisciplinary team discussion or even an interprofessional environment may be necessary.

#### Metacognition

As seen before, metacognition, part of the reflective mind (type 2a), is necessary for both type 1 and type 2 thinking [44]. Metacognition has been defined as ‘thinking about thinking’ or as the possibility to monitor and influence cognitive processes [45]. Metacognition provides feedback on the primary response, strengthening the response if it is correct so that it will be used in the future, as was also seen in the case [39]. Otherwise, if the response was not correct, it would be used less often. Metacognition can also activate type 2 thinking [46], monitoring the analytical thinking process, and is necessary to validate or reject the final choice [39]. Kahneman stated that if a physician sees an immediate effect of his/her work, then it will be easier to create appropriate patterns to recognize in a next similar situation because of this direct feedback [32]. Based on metacognition, cognitive forcing strategies provide a formal cognitive debiasing approach to deal with pitfalls in clinical reasoning. It can help to prevent errors, by identifying scenarios in which error is likely to occur [47].

#### Experienced and inexperienced physicians

Although type 1 thinking is always activated first, it is more likely to be correct among experienced prescribers [48–50]. This was also seen in the case report, where the supervisor seemed to directly know which medication to start. However, it may also happen that ingrained patterns prevent them from recognizing when they need to think again. In the case report, was the supervisor’s suggestion the best for the patient or was there newer knowledge that would lead to another choice? Inexperienced prescribers typically use type 2 thinking because they lack sufficient experience with various treatments in different contexts – they need to think their choice through [39, 51]. However, even an experienced prescriber may be inexperienced in a new situation. Moreover, experience does not always lead to expertise. Expertise not only requires experience, but also the ability to recognize when a problem needs slow analytic thinking [39, 51].

#### Therapy scripts

As prescribers gain experience, they can also develop therapy scripts with the help of metacognition [39]. Therapy scripts, which are knowledge structures that guide development of a management plan, are formed on the basis of experience and can be activated when a similar situation happens [18, 52–54]. Experience can be informed by treatment results, guidelines and feedback from colleagues, among other factors. In the case report, the supervisor directly seemed to know what to do based on his own experience. However, it can be challenging to explain this to novices, because of the automatism of this choice [16]. A diagnosis activates several therapy scripts, one of which is selected [18, 52]. Abdoler and colleagues found that drug knowledge and patient characteristics were important determinants of the choice of which therapy script to use [41]. Therapy scripts need to be adapted regularly, because contextual factors, such as comorbidities or patient preferences, ensure that standard treatment is not always possible [52]. Cook et al. stated that the best scripts have the same general framework, which is then adapted based on the specific illness and the patient [55]. According to them, therapy scripts consist of six key features, namely (i) the problem to be solved; (ii) management options; (iii) preferences, values, and constraints; (iv) education needs; (v) interpersonal interactions; and (vi) encounter flow (timing and sequence of events such as teaching, additional diagnostic testing and decision making) [53]. The quality of the script is based on four attributes, namely, (i) script content (disease-specific knowledge), (ii) a logical sequence, (iii) flexibility (the physician’s capacity to tailor the management plan to the unique patient), and (iv) fluency (a smooth rhythm that indicated familiarity with the material and efficiently conveyed specific, essential information without repetition or digression) [53]. According to Custers, illness scripts consist of three main components, namely, the enabling conditions (the patient and contextual factors), the fault (underlying pathophysiological process, which is basically textbook knowledge), and the consequences (complaints, signs, and symptoms) [56]. Students and inexperienced physicians are theorized to have simplistic scripts, based on their knowledge of the fault, whereas experienced physicians have sophisticated scripts, with greater individual variation, based on their own experience and the enabling conditions [52, 53, 56–58]. However, this distinction is largely theoretical, as empirical evidence directly observing and measuring these script differences remains limited. The differences of the scripts between students and inexperienced physicians and experienced physicians can be seen in Table 2. It is hypothesized that unlike illness scripts, therapy scripts often require both type 1 and type 2 thinking, whereby type 1 thinking is involved in script activation and type 2 thinking in script selection and revision, especially when the physician is not familiar with the problem or the problem is complex [22, 53].

**Table 2:** Differences in scripts between inexperienced and experienced prescribers

Scripts of students or inexperienced prescribers	Scripts of experienced prescribers
Simplistic scripts	Sophisticated scripts
Rely on their knowledge of the fault	Rely on enabling conditions
Mostly general scripts	More individual variation
Mainly based on textbook knowledge	Mainly based on their own experience

### Different models of therapeutic reasoning

Because of the differences between therapeutic and diagnostic reasoning, several models of therapeutic reasoning have been suggested. The knowledge from these models has been incorporated into our model. Aspects from these models are incorporated, supplemented by new insights. In this section, we will first discuss the different models and then focus on the differences with our model.

First, in 1994, the WHO published the 6-step model for therapeutic reasoning and prescribing, (see Fig. 1) This model was based on a structured observational study in 1984 involving 500 patient consultations in the practices of 50 physicians. This showed, among other things, that almost all doctors had a standard therapy in mind for most conditions (step 3a), where they checked whether it was suitable for the patient in question (3b). The widely used 6-step model in CPT education describes in detail how students can learn these steps, but it does not explain how and why prescribers choose their therapy in step 3. This explanation should be included in this step when the WHO model is revised [15, 21].

Bissessur and colleagues published a hypothetical model for therapeutic reasoning, based on the dual process theory [18]. In this model, it is possible to switch between non-analytical and analytical (type 1 and 2 respectively) thinking during the reasoning process. In the model proposed by Mancuso and Rose, physicians assess different facts, called focal points, to reach a composite decision. This assessment is influenced by the physician’s knowledge and/or experience, which can explain differences in therapy choice [59]. Walker et al. described three different stages of therapeutic reasoning in pharmacy students. First, they gather information, then they analyze it, for example, to assess whether the problem matches the management plan, and lastly they articulate management options and make their final decision with metacognition involved in all stages [60]. Mertens and colleagues have studied cognitive processes in pharmacists, leading to an eight step model—problem and demand for care consideration, information collection, clinical reasoning, clinical judgment, shared decision-making, implementation, outcomes evaluation, and reflection—each step coming with their own cognitive processes [61]. Cook and colleagues proposed a model of therapeutic reasoning with four steps: (1) instantiation of a management script, (2) identification of options and explanation to the patient, (3) shared decision-making, and (4) ongoing monitoring and adjustment. In this model, the process occurs between individuals (for example, the physician–patient dialogue), and not only in the physician’s mind [55]. As a result of that, this reasoning process may be ineffective if the physician does not encourage patient autonomy, does not involve the patient in the decision-making process, and does not include their (underlying) preferences in the final decision [62]. Physicians also need to be aware that patients have their own cognitive scripts that guide their interactions with their physicians, which could also affect the therapeutic reasoning process, through shared decision-making [22, 62].

Our European model of therapeutic reasoning as presented earlier is based on various models as described in this paragraph, while also establishing a connection between clinical practice, therapeutic reasoning, and contextual learning. A unique aspect of this model is that it always begins with some form of type 1 thinking and differentiates between type 2a and 2b thinking, as made in cognitive psychology. This also provides insights for educational approaches.

### Errors and bias in therapeutic reasoning

Therapeutic reasoning, like other forms of reasoning, is susceptible to errors. As other models have indicated, type 1 thinking is often prone to bias. Bias is common in therapeutic decision making and can manifest in various forms such as availability bias, impact bias, loss/gain framing effect, commission bias, omission bias, order effects, and relative risk bias [63]. An example of bias, namely commission bias, concerning the tendency to prefer action over inaction, was described in the example case, where the resident decided to treat the patient for pyelonephritis instead of a urinary tract infection, just to ensure he would not cause any harm by missing a serious infection. This outweighed concerns about the harm caused by antibiotics [64, 65]. An overview of the most frequently mentioned biases including examples in therapeutic decision-making can be found in Table 3 [22]. However, some of these sources of bias have only been found in hypothetical studies [63].

**Table 3:** Common biases in therapeutic reasoning

**Bias**	**Explanation** *Example*
Availability bias [63]	Making a decision based on an example, although it is not the most suitable one *Prescribing the same drug as for a similar patient with the same condition, although there are other patient characteristics that require a different choice*
Impact bias [63]	Overestimate and/or underestimate the effect of your choice *A physician considers antibiotic prescribing causing resistance an important clinical problem, but says that it is caused by physicians from other specialties and that they have to solve it*
Loss/gain framing effect [63]	Decision based on whether outcomes are presented as potential gains or losses, often favoring risk avoiding when it comes to gains and a willingness to take risks when facing losses *A physician decides to prescribe a specific drug based on the assumption that 90/100 patients will not have any side effects or to not prescribe that drug based on the assumption that 10/100 patients will experience a side effect that causes them to stop taking the medication*
Commission bias [64, 65]	Tendency to prefer action over inaction *Concern about missing an infection outweighs concern about serious antibiotic-induced harm such as Clostrioides difficile*
Omission bias [63]	Tendency toward inaction over action *Not treating a patient with antibiotics because of fear of resistance or other antibiotic-induced harm*
Order effects [63]	Sequence has impact on your choice, i.e. early alphabet options in a ranking are more likely to be chosen *Electronic prescribing from a list in alphabetical order, although another drug is more patient-friendly*
Relative risk bias [63]	Bias towards the relative effect – comparing risks between groups – over the absolute effect – the actual difference in risk *Tendency to look at relative risk reduction (e.g. a 50% reduction in risk) rather than the absolute risk reduction (e.g. a reduction from 2 to 1%) when making a therapy choice*
Premature closure [60]	An individual may accept a recommendation without considering other options *Prescribing the first drug that comes to mind instead of considering other drugs*
Belief bias [66]	Defending the type 1 decision if they believe the answer is correct, instead of analytically reconsidering it *A supervisor gives non evidence based arguments to defend his/her choice, instead of reconsidering the choice using analytical processes*

While bias in type 1 thinking is frequently addressed in reasoning models, it is important to note that bias and errors can occur during other stages of reasoning as well. This can among other things occur when prescribers do not recognize the need to switch to type 2b reasoning [16, 32, 35, 60]. Stanovich has described default to the autonomous mind (not activating analytical thinking) as the most significant thinking disposition [35]. This can occur through different mechanisms. On one hand, bias, such as premature closure, where a physician accepts a treatment recommendation without considering other options, can result in a failure to detect conflict, a discrepancy between signs and symptoms and the proposed treatment [60]. On the other hand, owing to limited working memory, our brains tend to prefer the easiest option, meaning that choices are often based on recognition of an option rather than considering its details [32]. This was seen in the case where the resident had to make his treatment choice quickly because he needed to see another patient, which would require working memory and a moment of reflection. In the end, he chose his type 1 response instead of reconsidering this. This tendency could partly explain why students frequently make their therapy choice the easy way – by following the example set by their teachers, which could lead to suboptimal therapy choices [16]. In such cases, the brain tends to opt for the cognitive ease of type 1 thinking [36]. While this often works well and in some cases the type 1 response (gut feeling or intuition) may be even more sufficient when it aligns with underlying reasons for caution [67, 68], individuals must remain sufficiently alert to situations where it is inadequate and where something needs to be changed. Failure to recognize when this is necessary is also one of the most common thinking errors [35]. However, type 2 reasoning does not always lead directly to the best treatment plan. This can occur due to override failure, where an individual recognizes the need for more analytical thinking but is unable to engage in it, for example due to limited cognitive capacity. Other potential issues include mindware gaps, where there is insufficient knowledge, or contaminated mindware, where the knowledge is incorrect. Additionally, serial associative cognition with a focal bias may cause someone to consider only a limited range of options, leading to suboptimal decision-making [35].

Other pitfalls in therapeutic reasoning, which can occur in both type 1 and type 2 reasoning, are vague or restricted care plans, failure to ascertain patient preferences, failure to follow cues of the patient, no shared-decision making, and no confirmation of understanding and commitment by the patient [69]. In addition, novices are often uncertain about their diagnosis, which makes it difficult to draw up a treatment plan and start treatment. Consciously or unconsciously, they are more worried about giving the wrong treatment than they are about not starting treatment [70].

## Implications for practice

### Implications for teaching therapeutic reasoning

There are strategies to help students develop clinical reasoning skills, although many of these strategies focus on diagnostic reasoning. These strategies influence one or more of the different thinking steps in the European model of therapeutic reasoning. While there are notable similarities that make these strategies based on diagnostic reasoning applicable, it is important to emphasize that empirical research is needed to determine whether they are equally effective for developing therapeutic reasoning skills. Nevertheless, until further research is available, these teaching strategies can serve as a useful framework. By using these teaching strategies for students, the aim is to provide them guidance to use the learned skills to become better prescribers.

Therapy scripts play an important role in type 1 reasoning. The differences between the scripts of experienced prescribers and residents suggests that it might be beneficial to focus on context rather than solely on textbook knowledge [71–73]. Seeing patients might help students develop context-rich therapy scripts at an early stage, instead of only case-based training (often based on textbook-based examples). Additionally, it would be particularly helpful for students to follow patient outcomes after their treatment decisions, which would enable them to reflect on their choices, learn from mistakes, and form richer scripts [32]. A greater exposure of students to more context (patient-related factors etc.) may lead to the development of better therapy scripts and make students more receptive to conflict detection, because they are better able to understand the necessity of changing their mind in some cases. This enables students to be more critical of their type 1 response and to recognize earlier when it is necessary to switch to type 2 reasoning. Moreover, students need to be aware of prescribing pitfalls, such as comorbidities or drug-drug interactions. Case-based teaching and assessment methods, such as those based on the WHO 6-step approach, can improve students’ ability to recognize these pitfalls in the future [15, 73, 74].

A person needs to be aware of the necessity to activate type 2a reasoning, which requires intrinsic motivation. Teachers should create circumstances that foster this. According to Deci’s self-determination theory, autonomy, competence, and relatedness are important for intrinsic motivation [75]. This may also lead to better memory formation because of different neural processes, such as stimulation of the dopaminergic systems and activity in brain networks for salience detection, attentional control and self-referential cognition [76, 77]. For example, participation in student-run clinics, where undergraduate students in the pre-clinical phase can treat real patients, or in case-based discussions during undergraduate clinical clerkships can promote high intrinsic motivation to learn how to prescribe effectively [78]. This is because students can gradually gain autonomy for treating real patients in a safe, practical setting within student-run clinics, by working collaboratively in a team [79]. Additionally, involvement in student-run clinics or other clinical settings provides exposure to a variety of cases, leading to more therapy scripts [78, 80]. However, it is important to keep the zone of proximal development in mind [81] – the task should be challenging but doable with help from others, otherwise it could diminish motivation and self-confidence. Groups of students could work together, each contributing their own knowledge and experience. Also, acknowledging that uncertainty is to be accepted in medicine and several therapeutic options may be appropriate are key aspects for learners and must be considered by medical educators in organizing their teaching [82]. Therefore, appropriate assessment methods, such as case-based assessments like observed structured clinical examination (OSCEs), are more applicable to asseses therapeutic reasoning, where choices can be explained, rather than multiple-choice exam questions with only one correct answer [83]. Next to that, also experienced prescribers recognize when they should take more time and switch to type 2 thinking when drawing up a treatment plan and need to encourage this awareness when seeking to improve the therapeutic reasoning of students. Additionally, experienced prescribers should explicitly explain their reasoning to check their own thinking and help less experienced learners develop more detailed scripts while avoiding cognitive biases. To help teachers to achieve these skills, also for example teach the teacher courses may be helpful [82].

Type 2 reasoning might be facilitated if students’ thought processes are structured, for example, by using the WHO 6-step or a management script template. The first steps of the WHO 6-step can also help to activate a therapy script (type 1), whereby the suitability for the patient can be considered in the next steps (as part of type 2 thinking). While the WHO 6-step can be used as cognitive forcing strategy [47], it is essential that students also have enough knowledge to weigh the different treatment options, so there still needs to be enough opportunity to gain this knowledge. Next to that, students must also know how to critically weight different options, for example by getting informed about the correct use of guidelines and evidence based medicine. Research showed that difficulties finding and using information from clinical guidelines contribute to medication errors [84].

Strategies to stimulate critical thinking and metacognition in general have been developed for use in case-based teaching, but these strategies have barely been studied in the context of therapeutic reasoning. Potentially relevant strategies are listed in Table 4 along with their primary area of impact, but some of these strategies still need to be tested and, if necessary, adjusted for therapeutic reasoning [46, 85–93]. The strategies can add structure (type 2), slow down the reasoning process and help to identify scenarios in which error is likely to occur, calling for switching from type 1 to type 2), and help students to reflect on their reasoning process (improving metacognition). CPT teachers must be cognizant of these various steps and should consider incorporating them in their educational approaches to develop a comprehensive understanding of successful therapeutic reasoning strategies. This is crucial because each step is essential and should therefore be cultivated through education. Teaching resilience in switching from type 1 to type 2 is important for both students and teachers [22].

**Table 4:** Clinical reasoning teaching strategies [46, 85–93]

Strategy	Short explanation
*In general*	
Case-based teaching	For example, by solving written clinical cases or engaging in role play (with or without actors portraying patients). Case-based teaching forms the foundation of other teaching strategies Case-based teaching enables students to apply their knowledge in (simulated) clinical practice. This approach can help them develop richer, context-based therapy scripts, and potentially lead to greater motivation compared with traditional lectures
Case-based assessment (i.e. Objective Structured Clinical Examination (OSCE))	Stimulates students to use their knowledge in (simulated) clinical practice, which might facilitate conflict detection. In addition, it provides students with feedback that might stimulate their metacognitive skills
Mechanism maps	Making visual maps, based on causality between concepts
Student-run clinic	Train prescribing skills grounded in a real-life context to provide students with early clinical experience and responsibility. This may result in a high level of intrinsic motivation and richer therapy scripts
*Stimulating metacognition*	
Time-out (i.e., diagnostic time-out or management pause)	Time out during reasoning process to evaluate the reasoning process (reflection-in-action) stimulates type 2 thinking and metacognition Specific questions during this pause are: (i) why are we choosing this intervention for this patient?; (ii) what are the potential downsides?; (iii) what are potential alternatives and why are we not choosing them?; (iv) have we asked the patient for their perspective?
TWED	Treat (What are the threatening conditions in this patient?) Wrong (What if I am wrong? What else could it be?) Evidence (Do I have sufficient evidence for or to exclude this diagnosis?) Dispositional factors (What are the environmental and emotional dispositions influencing my decision?)
Deliberate reflection	Approach to review a clinical case systematically (read the case, what are pro’s and con’s, are there any other possibilities)
Guided reflection	Real-time feedback on reasoning during a discussion, i.e. Why? What can also cause this?
Reflective writing	Stimulates metacognition by stepping back, reviewing thoughts, goals and actions, and recognizing how your perspectives, motives, and emotions affect your conduct (reflection-on-action)
Equity reflection	Reflection based on two main questions and follow-up questions 1) Are we deviating in any way from the standard of care in this situation? In what way, why and can we do something differently? 2) If you were being discharged in the same situation as this patient, is there anything you would want to be done differently than our present plan?
*Stimulating structure*	
One Minute Preceptor	1. Summarize the case; 2. Get a commitment; 3. Probe underlying understanding; 4. Reinforce what was done well; 5. Teach General Rules; 6. Correct errors
SNAPPS	1. Summarize relevant patient history and findings; 2. Narrow the differential diagnosis; 3. Analyze the differential diagnosis; 4. Probe the preceptor about uncertainties; 5. Plan management; 6. Select case-related issues for self-study
WHO 6-step	1. Define the patient’s problem; 2. Specify the therapeutic objective; 3. Choose your standard treatment and verify the suitability of your treatment; 4. Start treatment; 5. Give information, instructions, and warnings; 6. Monitor (and stop?) treatment

### Future perspectives

While the findings of this article certainly provide direction, there is much we do not yet know that requires further research, especially with regard to the suitability of the theories from cognitive psychology and diagnostic reasoning. Our European model for therapeutic reasoning is partly based on models for diagnostic reasoning, but it needs to be tested in practice because there are significant differences between diagnostic and therapeutic reasoning. Furthermore, it would be valuable to determine whether the therapeutic reasoning of different prescribers and non-prescribers, such as physicians, physician assistants, dentists, pharmacists, and advanced nurse practitioners, is similar and, if not, how differences should be appropriately addressed. While there have been individual studies on the thinking process of different prescribers or specific situations [61, 94], comparative studies examining their cognitive processes are still lacking.

Recognizing when to adopt type 2 thinking is an important principle of therapeutic reasoning and should be included in CPT education and supervision. Do experienced physicians use predominantly type 1 reasoning while inexperienced physicians rely more on type 2 reasoning more often for therapeutic reasoning, as they do for diagnostic reasoning? The complexity of therapeutic reasoning may mean that type 2 reasoning is used more often than type 1 reasoning by both inexperienced and experienced prescribers. Although it is assumed that therapy scripts are formed and used in almost the same way as illness scripts, it remains unclear how these work and how rich, context-based, therapy scripts, can be created as rapidly and efficiently as possible. Next to that, understanding formal models may also contribute to the development of therapeutic scripts by clarifying which information is used and how it is weighted.

More research is needed into whether strategies to teach clinical reasoning are applicable to therapeutic reasoning and how this can be optimized for both pre-graduate students and non-experienced doctors. Because of differences between experienced and inexperienced prescribers, it is essential to optimize the interaction between them to stimulate the therapeutic reasoning process, for example during supervision moments. Research must show how this can be optimized. Failure to recognize a conflict between a type 1 response and available clinical information is an important source of error, and so more needs to be learned about how to recognize the need to switch to type 2 thinking. Next to that, teachers must be able to recognize when their students’ type 1 reactions are inadequate so that they can provide useful feedback. Given the increasing prominence of artificial intelligence, it is pertinent to examine how it can facilitate therapeutic decision-making, both in clinical practice and as an educational tool [22].

## Conclusion

Most theories of clinical reasoning, both diagnostic and therapeutic reasoning, have been derived from cognitive psychology. However, because of differences between diagnostic reasoning and therapeutic reasoning, it is uncertain whether these theories can be applied to therapeutic reasoning as well [95]. One of the most important models is the dual process theory. This theory distinguishes between type 1 thinking, which is non-analytical and based on pattern recognition with the use of scripts, and type 2 thinking, which is analytical and takes more effort. Because type 2 thinking uses working memory, prescribers tend to make type 1 decisions unconsciously most of the time, especially when they are busy or tired. There always seems to be input from type 1 thinking, which may or may not be recognized by type 2 thinking. As Stanovich described, the most important thinking disposition is default to the autonomous mind – type 2 thinking is not activated if there is no conflict between the type 1 response and other patient information/findings. There must be some awareness and/or motivation for conflict detection, otherwise this will not happen. Metacognition, by which the reasoning process is monitored, is generally not included in the earlier models of therapeutic reasoning [18, 59, 60]. However, in our European model metacognition is involved in a large part of the model, as a monitor of the reasoning process. Therefore, it is essential to develop metacognitive skills, including the ability to discern when and how to transition between type 1 and type 2 thinking. Therefore, greater emphasis should be placed on this aspect during CPT teaching. A revised European model of therapeutic reasoning has been developed, and the interested reader can find more extensive information about its theoretical basis and the theories described in diagnostic reasoning and cognitive psychology in our report [22]. Further research is required to fully understand and optimize the therapeutic reasoning process. By advancing our understanding of therapeutic reasoning processes, we can also improve algorithms and create optimized decision support systems, which, given the advances in artificial intelligence, are poised to play an increasingly pivotal role in future prescribing practices.

## Data Availability

No datasets were generated or analysed during the current study.
